# Role of astrocytes in sleep deprivation: accomplices, resisters, or bystanders?

**DOI:** 10.3389/fncel.2023.1188306

**Published:** 2023-06-26

**Authors:** Mengxin Que, Yujuan Li, Xuan Wang, Gaofeng Zhan, Xiaoxiao Luo, Zhiqiang Zhou

**Affiliations:** ^1^Hubei Key Laboratory of Geriatric Anesthesia and Perioperative Brain Health, Department of Anesthesiology, Tongji Medical College, Wuhan Clinical Research Center for Geriatric Anesthesia, Tongji Hospital, Huazhong University of Science and Technology, Wuhan, China; ^2^Department of Oncology, Tongji Medical College, Tongji Hospital, Huazhong University of Science and Technology, Wuhan, China

**Keywords:** astrocytes, sleep deprivation, glymphatic system, neuroinflammation, sleep deprivation comorbidity

## Abstract

Sleep plays an essential role in all studied animals with a nervous system. However, sleep deprivation leads to various pathological changes and neurobehavioral problems. Astrocytes are the most abundant cells in the brain and are involved in various important functions, including neurotransmitter and ion homeostasis, synaptic and neuronal modulation, and blood–brain barrier maintenance; furthermore, they are associated with numerous neurodegenerative diseases, pain, and mood disorders. Moreover, astrocytes are increasingly being recognized as vital contributors to the regulation of sleep-wake cycles, both locally and in specific neural circuits. In this review, we begin by describing the role of astrocytes in regulating sleep and circadian rhythms, focusing on: (i) neuronal activity; (ii) metabolism; (iii) the glymphatic system; (iv) neuroinflammation; and (v) astrocyte–microglia cross-talk. Moreover, we review the role of astrocytes in sleep deprivation comorbidities and sleep deprivation-related brain disorders. Finally, we discuss potential interventions targeting astrocytes to prevent or treat sleep deprivation-related brain disorders. Pursuing these questions would pave the way for a deeper understanding of the cellular and neural mechanisms underlying sleep deprivation-comorbid brain disorders.

## 1. Introduction

Humans spend approximately one-third of their lives sleeping, which is known to be a necessary and conserved function throughout mammalian life (Krueger et al., 2016; Ingiosi et al., 2020). Normal sleep is divided into two phases: non-rapid eye movement (NREM) sleep and rapid eye movement (REM) sleep. Several processes have been linked to sleep, including cognitive processes such as memory consolidation and emotional control, as well as physiological processes such as hematological system modulation, tissue regeneration, cellular metabolism, endocrine regulation, and even atherosclerosis prevention (Spiegel et al., 1999; Tononi and Cirelli, 2014; Palmer and Alfano, 2017; Elkhenany et al., 2018; Raven et al., 2018; McAlpine et al., 2019). However, sleep disorders are associate with pain and various central nervous system (CNS) diseases, including mood disorders, psychiatric disorders, and neurodegenerative disorders such as Alzheimer’s disease (AD), Parkinson’s disease (PD), and Huntington’s disease (HD) (Tang et al., 2021; Winer et al., 2021; Nassan and Videnovic, 2022; Kunz et al., 2023; Stankeviciute et al., 2023).

Sleep deprivation (SD) is defined as prolonged periods of time without sleep. Today, SD is no longer a minor group condition but has evolved into an epidemic that is a major social and public health concern. Rising work demands, increasing prevalence of shift work, abuse of drugs that may have sleep-suppressing side effects, and increased use of artificial light-emitting devices contribute to sleep-related issues. In the context of chronic insomnia disorder, Zhang et al. (2018) demonstrated that inadequate sleep damaged cerebral microstructure, affecting astrocytes, neurons, and neuronal terminals; consequently, the damage resulted in impaired cognition, alertness, hippocampal connections, and episodic memory (Ikegami et al., 2009; Boardman et al., 2018; Chai et al., 2020; Smith et al., 2020; Ochab et al., 2021). It was once believed that recovery sleep would be accompanied by cognitive restoration; however, a growing body of research has cast doubt on this idea (Axelsson et al., 2008; Pejovic et al., 2013; van Straten et al., 2018). Accordingly, a list of abnormal changes in the brain after SD should be carefully evaluated.

Astrocytes are highly heterogeneous brain cells; apart from numerous subtle morphological changes, they have a ramified structure and intricate arborization. Reactive astrocytes might be generally divided into two subtypes: a neuroprotective phenotype (A2) and a neurotoxic phenotype (A1) (Fan and Huo, 2021). Liddelow et al. (2017) in their study, identified that lipopolysaccharide (LPS)-stimulated microglia produced differentiation factors, including tumor necrosis factor, interleukin-1α, and complement component 1q to increase an A1 astrocyte phenotype. Conversely, by analyzing samples from ischemic brain, they assigned A2 astrocytes neuroprotective activity (Liddelow et al., 2017). Astrocytes engage in synaptic pruning (Lee et al., 2021) and phagocytosis of damaged or dead cells (Davis et al., 2014; Morizawa et al., 2017; Wan et al., 2022), thus contributing to the maintenance and prolongation of brain homeostasis (Damisah et al., 2020) and helping delay the progression of degenerative diseases, although the precise regulatory process involved in such waste/debris removal is significantly affected by aging.

Astrocytes play an important role as timekeepers of the hypothalamic suprachiasmatic nucleus, which is considered as the central pacemaker (Astiz et al., 2022), and the astrocyte cell-autonomous molecular clock can drive circles in the daily neuronal circuit (Hastings et al., 2023); moreover, they possess 55 unique sleep genes and 396 unique awake genes (Bellesi et al., 2015). Additionally, astrocytes serve various specific purposes during sleep and wakefulness, including information processing and cognitive consolidation (Pannasch and Rouach, 2013; Sardinha et al., 2017; Adamsky et al., 2018). Some astrocyte secretions increase sleep time or non-rapid eye movement slow-wave activity, which in turn affect astrocyte morphology and gene expression patterns (Frank, 2019). Using transcriptomic profiling experiments, researchers demonstrated that some astrocytes were state-dependent and that their metabolism and activities were mostly upregulated in the awake state. At the same time, sleep evoked the expression of a few specific genes to extend peripheral astrocytic processes, like *Cirp* and *Uba1* (Bellesi et al., 2015). Nevertheless, although a few breakthrough studies have shown that astrocytes are crucial for sleep regulation, the mechanisms underpinning their contribution to physiological sleep and SD comorbidities are still unclear.

Sleep deprivation hampers daily functions of astrocytic that protect neuronal homeostasis. However, the associations among SD and astrocytes and subsequent brain disorders remain poorly investigated. In this review, we discuss the current views on astrocytic contributions to physiological sleep, from molecular mechanisms to systematic manifestations in the case of SD. Subsequently, we discuss pathological ailments such as stroke, epilepsy, and neurodegenerative diseases, usually worsened by coexisting sleep deprivation. Finally, we elaborate on the potential mechanisms by which astrocytes contribute to SD-related comorbidities and discuss some feasible strategies and possible treatments for the various neural consequences. These findings are expected to shed light on potential therapeutic strategies for managing sleep loss and its associated comorbidities.

## 2. Role of astrocyte in normal sleep and sleep deprivation

**FIGURE 1:**
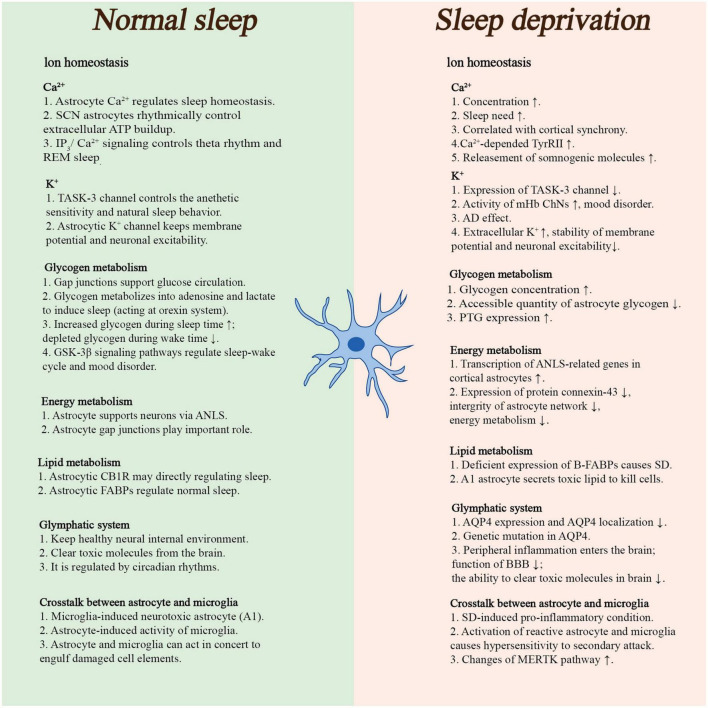
This panel briefly summarized the roles of astrocyte in physiological sleep and sleep deprivation. (ATP, adenosine triphosphate; AD, Alzheimer’s disease; ANLS, astrocyte–neuron lactate shuttle; CB1R, type 1 cannabinoid receptors; FABPs, fatty acid-binding proteins; MERTK, tyrosine-protein kinase MER; mHb ChNs, cholinergic output neurons in the medial habenula complex; PTG, protein targeting to glycogen; REM sleep, rapid eye movement sleep; SCN, suprachiasmatic nucleus; SD, sleep deprivation; BBB, blood–brain barrier).

Astrocytes participate in various physiological activities in the brain, from ion balance to metabolism. As important components of the energy metabolism process in the brain, astrocytes work with neurons to significantly influence overall brain activity. Additionally, astrocyte–microglia cross-talk has been linked to several physiological functions, including immunological functions. Astrocytes are also involved in the control of cerebral blood flow through neurovascular coupling, regulating synaptic activity and plasticity, encircling the synapses of other neurons, helping to form the blood–brain barrier (BBB), which ensures brain homeostasis, and releasing vasoactive substances that cause arteriole dilation in highly active neural regions. In 1895, Cajal hypothesized that astrocytes control sleep by extending their dendrites into synapses during sleep and retracting them during wakefulness (García-Marín et al., 2007). Although this hypothesis was later proven inaccurate (Bellesi et al., 2015), recent studies have highlighted the importance of astrocytes in modulating sleep (Halassa et al., 2009; Jackson et al., 2020). The detailed regulation of astrocytes on sleep would be discussed in the following section and the summary information was outlined in Figure 1.

### 2.1. Neuronal activity

Through the release of gliotransmitters [adenosine, glutamate, gamma-aminobutyric acid (GABA), glycine, D-serine, lactate, and various cytokines], astrocytes play a significant role in regulating neuronal activity and synaptic transmission (Araque et al., 2014; Sahlender et al., 2014; Yoon and Lee, 2014; Mosienko et al., 2015; Petrelli and Bezzi, 2016; Bonvento and Bolaños, 2021; Park et al., 2022), which can synchronize neuronal activity in the different brain regions, such as the hippocampus, posterior hypothalamus, and cortex. Ion chemical signals in astrocytes are also correlated with neuronal synchrony, and synchronization is important for generating slow-wave activity and sleep (Garofalo et al., 2020). The characteristic electroencephalography EEG pattern of wakefulness can be rapidly induced in sleeping mice by manipulating the extracellular ion content (Ding et al., 2016).

#### 2.1.1. Astrocytic somnogenic molecules

##### 2.1.1.1. Adenosine

Adenosine is produced following a long period of wakefulness to regulate sleep homeostasis (Porkka-Heiskanen et al., 1997). Theoretically, gliotransmission can be switched on or off *in vivo* when paired with an astrocyte-specific inducible mechanism (the Tet-off system) (Morozov et al., 2003; Pascual et al., 2005). Pascual et al. (2005) showed that the selective expression of a dominant-negative soluble N-ethylmaleimide-sensitive protein receptor (dnSNARE) in astrocytes decreases the extracellular build-up of adenosine both *in situ* and *in vivo*. Moreover, adenosine builds up while awake, but extracellular adenosine concentration does not increase during SD, indicating that adenosine regulation during sleep and wakefulness is complex and that SD might also have a direct impact on adenosine tone (Zeitzer et al., 2006; Clasadonte et al., 2017). Using molecular genetics techniques, researchers discovered that astrocytes release adenosine triphosphate (ATP) as the source of extracellular adenosine via astrocytic adenosine kinase (ADK) (Bjorness et al., 2016) to act on synaptic A1 receptors of neurons in sleep-wakefulness-related neural regions; one example is the perifornical-lateral hypothalamic area (Alam et al., 2009), which provides highly region-specific negative feedback for inhibition or suppression of neuronal activation, contributing to circadian rhythmicity and increased NREM sleep, decreasing fear memory, and protecting the brain from excessive activation in healthy as well as certain disease states (Halassa et al., 2009; Badimon et al., 2020; Li et al., 2020). Moreover, ADK expression in astrocytes is associated with astrogliosis and synaptic transmission modulation, and it may also play a role in neuroinflammation (Garofalo et al., 2020). ADK is also involved in several neurotransmitter pathways, sleep regulation, and the generation of EEG oscillations (Palchykova et al., 2010). Furthermore, activation of adenosine A(2A) receptors activates basal forebrain glutamatergic, hypothalamic GABAergic, and striatal parvalbumin neurons, all of which play a role in improving sleep (Kumar et al., 2013; Yuan et al., 2017; Peng et al., 2020). Using pharmacological and genetic approaches, Jagannath et al. (2021) showed that adenosine regulates the circadian clock genes *Per1* and *Per2*, which affect circadian processes by activating adenosine A(1)/adenosine A(2A) receptors via the Ca^2+^-ERK-AP-1 and CREB/CRTC1-CRE pathways.

Peng et al. (2023) found that calcium activity in astrocytes from the basal forebrain (BF), a crucial regulatory region for sleep and wake behavior, can bi-directionally regulate sleep-wake behavior. Notably, this regulation occurs independently of extracellular adenosine signaling, challenging the previous understanding that adenosine from astrocytes is the sole contributor to extracellular adenosine levels (Peng et al., 2023). Calcium activity of BF astrocytes was activated and inhibited by the chemogenetic method and conditional knockout of IP_3_R2, respectively. It was found that activating the calcium activity of BF astrocytes caused NREM sleep disruption and reduced REM sleep duration, indicating worse sleep quality and higher levels of alertness. Moreover, the arousal level in mice was considerably decreased by inhibiting the calcium activity of BF astrocytes. Furthermore, Peng et al. (2023) demonstrated that in the BF, astrocyte calcium activity was primarily induced by neural activity rather than neuromodulatory signals like noradrenaline or acetylcholine. Through chemogenetic activation of astrocytes’ calcium activity in the BF, astrocytes decreased the consolidation of NREM sleep by amplifying the activation of the GABAergic neurons, which are more active during wakefulness than during sleep (Hassani et al., 2009), increasing persistent inhibition in the BF neural network. Thus, this study offers novel insights into the role and mechanism of astrocytes in sleep-wake regulation.

##### 2.1.1.2. Glutamate

Astrocytes are the only type of neural cells to express pyruvate carboxylase, providing extra glutamate to the brain by converting glucose into glutamate (Bélanger et al., 2011). By regulating synaptic activation and neuronal excitability via glutamate release, astrocytes support several physiological functions, including sleep homeostasis and memory consolidation (Pal, 2018). Astrocytes have long been known to possess the ability to release glutamate in response to prostaglandins via a Ca^2+^-signal-dependent pathway (Bezzi et al., 1998). Subsequently, it was demonstrated that optogenetically reactive astrocytes increase extracellular glutamate concentration, increasing REM and NREM sleep duration (Pelluru et al., 2016; Poskanzer and Yuste, 2016). Astrocytes may also remove glutamate from synaptic clefts in addition to releasing it. Glutamate is primarily absorbed by glutamate transporter 1 (GLT1) (a high-affinity glutamate transporter) and glutamate aspartate transporter (Bak et al., 2006). GLT1 has been reported to react differently in wake-promoting orexin neurons and sleep-promoting melanin-concentrating hormone (MCH) neurons depending on differences in sleep needs (Briggs et al., 2018); moreover, it can regulate the activation of glutamate receptors in astrocyte–neuron circuits, contributing to sleep normalization. Specifically, compared to rest time, SD enhanced perisomatic GLT1 apposition linked with sleep-promoting MCH neurons and reduced GLT1 apposition related to wake-promoting orexin neurons in the lateral hypothalamus. These findings indicate that astrocytes can make subtle adjustments in two oppositely functioning neuronal units associated with the sleep-wake cycle via glutamate transport, which could be a significant intervention prospect for treating sleep loss. Interestingly, astrocytic coverage in the synaptic cleft decreases during sleep—a phenomenon linked to neuronal synchronization during NREM sleep—and increases during awakening (Bellesi et al., 2015).

##### 2.1.1.3. Pro-inflammatory cytokines

Astrocyte-mediated inflammation is another important mechanism involved in the regulation of sleep. Astrocytic tumor necrosis factor-alpha (TNF-α), a pro-inflammatory cytokine, is an important sleep regulator. Several studies have shown that astrocyte-derived cytokines, including TNF-α and interleukin (IL)-1, support sleep and immunity (Krueger et al., 2011; Olivadoti et al., 2011; Blum et al., 2021), even though the function of TNF-α in sleep remains debatable (Szentirmai and Kapás, 2019). IL-6 can enhance adenosine A(1) receptor mRNA expression and signaling in astrocytes (Biber et al., 2001), and as mentioned above, adenosine is an established inhibitory neuromodulator that supports sleep homeostasis. With an emphasis on the sphingosine kinase 1/mitogen-activated protein kinase/protein kinase B (Akt) pathway, astrocytic aquaporin 4 (AQP4) is involved in the release of pro-inflamatmory cytokines (Dai et al., 2018). This suggests another astrocytic molecule that may play a role in regulating sleep normalization.

##### 2.1.1.4. γ-aminobutyric acid

It is well-established that γ-aminobutyric acid (GABA) is a primary inhibitory neurotransmitter that promotes longer sleep duration and regulates sleep in a conserved manner. It has been reported that somatostatin-expressing interneuron-derived GABA can mediate Ca^2+^ elevation in astrocytes, revealing an astrocytic, non-neuronal component of GABA-related inhibitory circuits (Mariotti et al., 2018). Concomitantly, Ca^2+^ elevation can also evoke gliotransmitter glutamate release promoting sleep (Toppila et al., 1997; Xu et al., 2015). As GABA is known to have a significant and conserved function in regulating sleep, GABAergic tone should be strictly regulated in sleep circuits. Recently, the astrocytic GABA transporter has been shown to reduce GABAergic tone, leading to longer sleep latency and sleep homeostasis disruption, while the hypomorphic *gat33-1* mutant had the opposite effect (Chaturvedi et al., 2022).

##### 2.1.1.5. Lactate

Lactate is a critical energy substrate for neurons and a signaling molecule that modulates neuronal excitability, plasticity, and memory consolidation (Magistretti and Allaman, 2018). The orexin system is well-known for controlling wakefulness and eating behavior (Tsujino and Sakurai, 2009), and lactate is important in controlling the orexin system (Parsons and Hirasawa, 2010). This suggests that lactate is also involved in regulating natural sleep. Glymphatic system astrocytic AQP4 can clear excessive brain lactate depending on sleep needs (Lundgaard et al., 2017). Noradrenaline, a potential waking signal that functions via activating astroglial β2-adrenergic receptors, is also reported to be linked to sleep–wakefulness mechanisms by affecting astroglial energy substrate metabolism to increase lactate production (Ingiosi and Frank, 2022).

##### 2.1.1.6. Others

Cis-oleamide (Cravatt et al., 1995), peptides such as urotensin II (Huitron-Resendiz et al., 2005), and anandamide are all involved in the sleep-induction process. Glial cells are known to process monoamines to maintain sleep homeostasis (Nall and Sehgal, 2014). According to a recent report, modulation of the alpha1-adrenergic receptors on astrocytes in the ventral periaqueductal gray may influence arousal (Porter-Stransky et al., 2019). In astrocytes, adrenaline is produced by monoamine oxidase, and a release of Ca^2+^ from the endoplasmic reticulum happens after the activation of phospholipase C by adrenaline (Novikova et al., 2020). Furthermore, this calcium activity in astrocytes was found to synchronize neurons and affect slow-wave activity. In Drosophila, arylalkylamine N-acetyltransferase 1 (AANAT1) can acetylate and inactivate monoamines; this also occurs in astrocytes and certain subsets of neurons in the adult brain. When AANAT1 was knocked down in astrocytes but not in neurons, flies in the knock-out group displayed increased sleep recovery the day following an overnight SD, demonstrating the significance of astrocytes in the regulation of monoamines and homeostatic sleep (Davla et al., 2020).

#### 2.1.2. Ion homeostasis

##### 2.1.2.1. Ca^2+^

**FIGURE 2:**
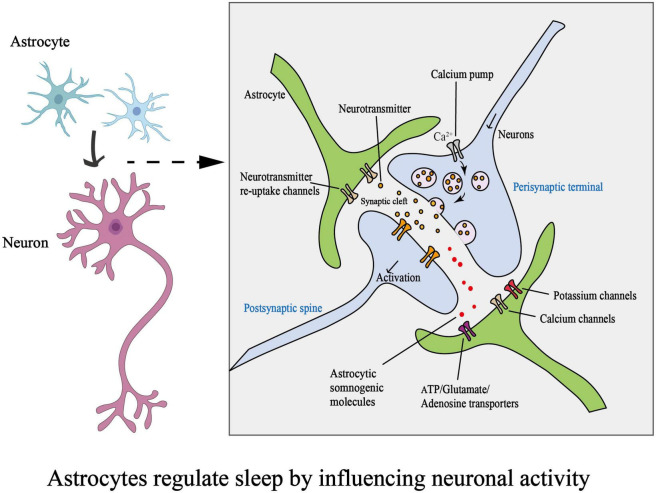
Schematic diagram on how astrocytes regulate sleep by influencing neuronal activity. (ATP, adenosine triphosphate).

The development of one- and two-photon microscopy and genetically encoded calcium indicators (GECIs) has allowed *in vivo* detection of astrocytic Ca^2+^ activity (Guerra-Gomes et al., 2017; Lim et al., 2021). Sleep regulates several astrocytic processes, including rhythmic intracellular Ca^2+^ signaling (Burkeen et al., 2011; Brancaccio et al., 2017). Communication between astrocytes and neurons can be seen through intracellular calcium elevation in astrocytes; this was recently demonstrated in *in vivo* studies (Bojarskaite et al., 2020; Peng et al., 2020). Moreover, Ca^2+^ elevation in astrocytes can stimulate them to release numerous chemical transmitters, such as glutamate, GABA (Parpura et al., 1994; Araque et al., 2001; Stout et al., 2002; Lee et al., 2010), ATP, and others (Marpegan et al., 2011; Svobodova et al., 2018; Figure 2).

Additionally, astrocytes are prevalent in the suprachiasmatic nucleus (SCN), where they control the build-up of extracellular ATP and connect ATP to intracellular Ca^2+^ signaling pathways (Burkeen et al., 2011). Burkeen et al. (2011) showed that in rat SCN2.2 cell cultures, rhythmic ATP build-up is accompanied by intracellular Ca^2+^ level fluctuations, and maximum extracellular ATP accumulation coincided with peak mitochondrial Ca^2+^ accumulation. Inositol trisphosphate (IP_3_)/Ca^2+^ signaling in astrocytes controls theta rhythm and REM sleep, and genetically lowering IP_3_ levels in astrocytes was reported to solely affect REM sleep (Foley et al., 2017). At typical slow-wave sleep levels, astrocytic Ca^2+^ signals are also critical (Bojarskaite et al., 2020). Bojarskaite et al. (2020) reported that NREM sleep was disrupted, and microarousals were more frequent in mice with a knocked-out IP_3_ receptor type 2 (IP_3_R2). There could be several reasons for this difference. First, although IP_3_R2 is the predominant receptor subtype, IP_3_R1 and IP_3_R3 also contribute to rapid Ca^2+^-related events in sleep processes (Tamamushi et al., 2012). IP_3_R2 is found in the soma and major branches, whereas IP_3_R1 is more likely to be localized in the peri-synaptic processes (Petravicz et al., 2014; Takano et al., 2020). Constitutive IP_3_R2 knock-out may not affect Ca^2+^ signaling in distal processes, which undergo the most dynamic changes during sleep-wake cycles. Second, the tissue distribution patterns of IP_3_ receptors in the body are complex. IP_3_R1 is mostly expressed in the CNS, whereas IP_3_R2 and IP_3_R3 are strongly expressed in the heart, pancreas, liver, and salivary glands (Hisatsune and Mikoshiba, 2017). Generally, the IP_3_ receptor can affect sleep-wake behavior; however, when it comes to specified sleep types, it is necessary to analyze the functions of IP_3_ receptor subtypes.

Humans have a compensatory mechanism to restore sleep homeostasis after SD. Using two-photon microscopy, Ingiosi et al. (2020) showed how astrocytes regulated sleep homeostasis after SD: astrocytic Ca^2+^ concentrations were lowest during the sleep phase but increased following SD in correlation with the demand for sleep. Additionally, after SD, less astrocyte synchronization occurred at both the network and single-cell levels during non-rapid eye movement sleep (Ingiosi et al., 2020). Another study on a Drosophila model indicated that specific astrocytic L-type Ca^2+^ channel-dependent Ca^2+^ signals increase to enhance sleep drive so that sleep demand can be met; moreover, increased levels of TyrRll (a monoaminergic receptor) in a Ca^2+^-dependent manner following SD can evoke further elevation of astrocytic Ca^2+^ levels and promote a positive feedback loop that contributes to sleep homeostasis (Blum et al., 2021).

##### 2.1.2.2. K^+^

Using an immunolabelling approach, Rusznák et al. (2004) demonstrated that astrocytes express high levels of two pore-domain acid-sensitive K^+^ channels (TASK-1 and TASK-3). TASK-3 channels are widely distributed in several brain regions, such as the hippocampus, cortex, and cerebellum, as well as in specific nuclei, including the locus coeruleus, paraventricular nucleus of the thalamus, and dorsal raphe nucleus (Talley et al., 2001). TASK-3 KO mice show apparent changes in both anesthetic sensitivity and natural sleep behavior, suggesting that TASK-3 plays a role in sleep-wake control (Pang et al., 2009); specifically, it is highly likely that some TASK-3 channel-targeted substances are involved in REM sleep homeostasis and the effects of antidepressant therapy (Borsotto et al., 2015). Furthermore, astrocytic K^+^ channels can clear superfluous extracellular K^+^ ions to modulate membrane potential and neuronal excitability, which is crucial for maintaining sleep homeostasis (Figure 2). Ding et al. (2016) showed that decreased extracellular K^+^ during sleep was coupled with increased volume in the extracellular space. The observed volume change is attributed to a decrease in astrocyte volume, indicating a shift in the proximity of astrocytes to synapses (McCauley et al., 2020).

Some studies have shown that SD reduces TASK-3 channel activity, which results in the activation of cholinergic output neurons in the medial habenula complex, a brain area associated with negative affect-related behaviors, including fear, anxiety, and stress (Ge et al., 2021), causing mood disorders. Longer sleep latency and worse sleep quality have also been observed in TASK-3-potassium-channel-knock-out animals (Pang et al., 2009). Additionally, reducing TASK-3 channel activity may promote AD progression (Borsotto et al., 2015). Neurological processes in HD (Tong et al., 2014; Khakh et al., 2017) and PD (Hu et al., 2019; Chen et al., 2021) are also linked to the clearance of excessive extracellular K^+^ via astrocytic K^+^ channels, which may alter membrane potential and neuronal excitability.

##### 2.1.2.3. G-protein-coupled receptors

Astrocytes possess an array of G-protein-coupled receptors (GPCRs) that help them sense neuronal sleep-wake signals by activating astrocytes’ calcium activity. Conversely, elevated calcium levels in astrocytes stimulate the release of neuroactive substances, including glutamate and GABA, which modulate synaptic activity via activating neuronal GPCRs. It has been reported that astrocytes in mice can regulate different features of NREM sleep via two different types of GPCRs: Gi-GPCRs, which are related to sleep depth, and Gq-GPCRs, which are related to sleep duration (Lin et al., 2019; Vaidyanathan et al., 2021). However, it remains unknown whether the same is applicable to humans.

### 2.2. Metabolism

#### 2.2.1. Glycogen metabolism

Astrocytes are the dominant source of glycogen in the brain in homeostasis and exhibit high glucose uptake and glycolytic rates. Gap junctions, which are formed by neighboring astrocytes at their distal processes with minimal overlap between the arbors of each cell, allow for the delivery of energy metabolites from arteries to distant neurons supporting neuronal activity; they also support glucose circulation throughout the brain (Rouach et al., 2008). As an energy resource, glycogen supports neural energy consumption (Belanger et al., 2011) and glutamatergic neurotransmission (Sickmann et al., 2009; Mozrzymas et al., 2011). Brain glycogen metabolism produces ATP (Zhang et al., 2021), and this metabolism has been extensively studied in neurons and astrocytes. In 1995 (Benington and Heller, 1995), researchers proposed that some of the extracellular sleep-inducing chemical adenosine derives from the release of ATP, which is replenished during sleep but depleted during waking (Bellesi et al., 2018). Namely, sleep can affect glycogen turnover in astrocytes. Glycogen metabolism can regulate sleep homeostasis. Overexpression of glycogen synthase kinase-3-beta (GSK-3β) also contributes to increased NREM sleep (Ahnaou and Drinkenburg, 2011). Some lactate derives from astrocytic glycogen (Itoh et al., 2003; Magistretti and Allaman, 2018), which may regulate sleep by controlling the orexin system (Parsons and Hirasawa, 2010).

The effects of SD on transcription may explain why SD increases glycogen concentration while decreasing the quantity of accessible astrocytic glycogen (Petit et al., 2021). Moreover, a decrease in astrocytic glycogen turnover leads to the collapse of various downstream cellular mechanisms. For instance, decreased glutamatergic neurotransmission and lactate production have detrimental effects on mood regulation and sleep homeostasis. There is evidence of a significant change in gene expression after SD, particularly of the glycogen metabolism-related scaffold protein known as protein targeting to glycogen (PTG) (Magistretti, 2006). The regulation of glycogen metabolism is mainly carried out by regulating the activities of glycogen synthase and glycogen phosphorylase. The PPP1c catalytic subunit of PTG colocalizes with specific glycogen-metabolizing enzymes; it dephosphorylates glycogen synthase and glycogen phosphorylase and increases glycogen synthesis flux. Overexpression of PTG in astrocytes increases glycogen accumulation by more than a hundred-fold, whereas knocking it down causes glycogen accumulation to drop by around 50% (Ruchti et al., 2016).

#### 2.2.2. Lipid metabolism

Lipids play a vital role in many physiological processes in the nervous system. Prostaglandin D2 (PGD2) is the most potent endogenous sleep-inducing substance (Hayaishi, 2002; Urade and Hayaishi, 2010) and functions via the PGD2-adenosine system (Urade and Hayaishi, 2011).

One of the primary regulatory systems in the brain is the endocannabinoid system (ECS), which has also been linked to sleep modification (Hanlon et al., 2015; Hodges and Ashpole, 2019; Kesner and Lovinger, 2020). Type 1 cannabinoid receptors are one of the most abundant GPCRs in the CNS and have gained considerable attention in recent years due to the variety of roles they play in astrocytic processes. For instance, through Ca^2+^ elevation, endocannabinoids can boost glutamate release from astrocytes and long-term potentiation of transmitter release at synapses, both of which contribute to neuronal synchronization and synaptic plasticity (Navarrete and Araque, 2010; Gómez-Gonzalo et al., 2015; Araque et al., 2017).

Fatty acid-binding proteins (FABPs) are associated with lipid and energy metabolism, inflammatory mechanisms, and cognitive dysfunctions (Furuhashi and Hotamisligil, 2008; Storch and Corsico, 2008; Teunissen et al., 2011; Jiang et al., 2021). Fabp7 is a kind of FABP expressed in mammalian astrocytes and neural progenitors. Fabp7 was found to play an indispensable role in normal sleep. Fabp7 can be regulated by the core circadian clock transcription factor BMAL1 (Furuhashi and Hotamisligil, 2008; Gerstner and Paschos, 2020). In both flies and humans, the presence of a missense mutation in astrocyte FABP7.T61M leads to fragmented sleep or SD (Gerstner et al., 2017).

Sleep deprivation can facilitate reactive astrocytic subtype transition in the context of mild neuroinflammation, various chronic neurodegenerative diseases, and brain injuries, leading to further inflammatory reactions. For example, neuroinflammation causes the induction of a neurotoxic reactive subtype, termed A1-type astrocytes, by microglial cytokine release (Liddelow et al., 2017), which can kill oligodendrocytes and neurons by secreting toxic lipids. According to one study, saturated lipids, rather than APOE and APOJ lipid granule proteins, mediate the toxicity induced by reactive astrocytes and are crucial for astrocyte-mediated toxicity. The specific knockout of the saturated lipid synthetase ELOVL1 (the elongation of very-long-chain fatty acids protein 1) in astrocytes reduced the toxicity of reactive astrocytes. These findings highlight the critical function of astrocytes in the response to CNS injury and neurodegenerative disorders, as well as of lipids in CNS signal transmission (Guttenplan et al., 2021).

#### 2.2.3. Energy metabolism: the glucose-lactate shuttle

Over the past decade, our thinking of neuroenergetics has changed from a neuron-centric viewpoint to a neuron-astrocyte cooperation perspective. While it comprises only 2% of body mass, the brain uses up to 25% of the body’s glucose and 20% of its oxygen. A range of glucose-derived energy substrates, including lactate, glutamate, glutamine, and pyruvate, are effectively utilized by brain cells. Astrocytes cover blood vessels with their endfeet, allowing them to take nutrients from the blood vessels into the astrocyte network and deliver them to distal neurons. The integrity of the astrocytic network correlates with the energy delivery chain in neurons and influences long-term neuronal plasticity (Murphy-Royal et al., 2020).

Specific gene expression profiles indicate that astrocytes can consume large amounts of glucose for anaerobic glycolysis, producing and releasing lactate into the extracellular space (Beard et al., 2021). The genes essential for controlling neuronal energy metabolism indicate that lactate is the preferred energy substrate of neurons (Herrero-Mendez et al., 2009). These complementary metabolic features provide a comprehensive energy supply chain for the brain. Astrocytic pyruvate carboxylase converts glucose into glutamate, and the specific glutamine synthase converts glutamate into glutamine (Bélanger et al., 2011). The astrocyte–neuron lactate shuttle (ANLS), essentially a glucose-lactic acid shuttle, is thought to be the main pathway for the interaction between neurons and astrocytes. The ANLS combines glutamate transporter activity with the conversion of glucose to lactate, which is then exported to the neurons for energy. Astrocytic lactate is essential for memory function, sleep regulation, and synaptic plasticity. Glutamate transporter activity is at the core of the ANLS. Glutamate transport within astrocytes is closely associated with increased intracellular Na^+^ concentration, which further activates Na^+^/K^+^-ATPase to stimulate glycolysis, prompting the utilization of glucose and the production of lactate (Gudkov et al., 2022). This suggests that astrocytes play a significant role in several brain energy metabolism processes critical for neurological function.

In mouse models, SD has been shown to increase the transcriptional regulation of ANLS-related genes in cortical astrocytes (Petit et al., 2013). Furthermore, patients with decreased sleep quality showed lower expression of biomarkers such as connexin-43 (a primary astrocytic gap junction protein), connexin-30, and AQP4 (Yang et al., 2022). The delivery of energy substrates may be hampered by decreased astrocyte gap junction coupling with dominant-negative connexin-43; moreover, the same outcome was observed when lactate outflow was impaired (Murphy-Royal et al., 2020). As neuronal long-term plasticity and energy metabolism can both be hampered by the disruption of the astrocyte network (Murphy-Royal et al., 2020), altering glycogen turnover and glutamatergic neurotransmission through the ANLS may be a potential mechanism for identifying and ameliorating the root causes of the vicious cycle of SD-related comorbidities.

### 2.3. Regulating the glymphatic system to influence the sleep-wake cycle

There is substantial evidence that cerebrospinal fluid (CSF) in the subarachnoid space can enter the brain through the perivascular space (PVS) and mix with the interstitial fluid (ISF) (Iliff et al., 2012). This system of extensive CSF-ISF exchange is also known as the glymphatic system; its essential function is maintaining a healthy internal environment and providing neural cells with optimal working conditions, as neural cells are highly sensitive to changes in the surrounding milieu.

A crucial regulatory mechanism to promote glymphatic fluid transfer is the polarization of AQP4 toward the vascular terminal foot. Moreover, changes in both astrocytes and blood vessels in the brain affect PVS regulation. Mestre et al. (2018) have shown that the strong activation of reactive astrocytes observed in F-8xFAD mice may lead to changes in PVS size, resulting in glymphatic flux reduction. The glymphatic system is found in the brains of rodents, pigs, and humans and plays an important role in clearing toxic molecules from the brain, including degenerative disease-related proteins—amyloid-β (Thal et al., 2002), α-synuclein (Ozansoy and Başak, 2013), and abnormal tau phosphorylation (Martin et al., 2013)—as well as inflammatory cytokines (Zbesko et al., 2018) and excessive lactate (Lundgaard et al., 2017). The lymphatic system depends substantially on the AQP4 water channels (Verkman et al., 2006; Mestre et al., 2018), which are located on astrocytic endfeet and are in contact with the vasculature, facilitating the flow of CSF from the PVS to the brain parenchyma. Moreover, the glymphatic system has been hypothesized to be more active during sleep, as there is an approximately 60% increase in the interstitial space during natural sleep or anesthesia compared to that in the conscious state (Xie et al., 2013). Recent studies have shown that the glymphatic system is regulated by circadian rhythms rather than the sleep-wake cycle (Cai et al., 2020; Hablitz et al., 2020). Even after the reversal of their ambient light-dark cycle, rats exhibited the same redistribution pattern as normal light-dark cycle rats, indicating that the glymphatic system may be affected by endogenous hormones and not just sleep/wake states (Cai et al., 2020). At the same time, the polarized distribution of astrocyte endfeet AQP4 is also related to different time periods of the day, and the ablation of the AQP4 gene effectively eliminates the circadian regulation of CSF distribution (Hablitz et al., 2020). Taken together, these findings prove the close association between the glymphatic system and sleep-wakefulness cycle.

Endothelial cells, pericytes, neurons, microglia, and astrocyte endfeet that surround an artery but are isolated from it by the basement membrane make up the neurovascular unit, which comprises cells intrinsic to the vessel wall. Astrocytes contribute to the BBB by generating the glia limitans and sending paracrine signals to endothelial cells, which are principally responsible for establishing and maintaining BBB integrity. The proteins connexin-43 and connexin-30 help to create plaques at gap junctions, which can connect astrocyte endfeet. Astrocytic gap junctions also play an important role in preserving the integrity and function of the glymphatic system. It has been reported that BBB permeability and AQP4 and connexin-30 arrangement on endfeet are determined by the connexin-43 carboxyl-terminal domain (Cibelli et al., 2021).

Another tight barrier, composed of tanycytes and astrocytes, at the circumventricular organs surrounding the brain ventricles may prevent blood-borne substances from easily migrating to nearby brain areas. Moreover, it has been shown that circumventricular organ astrocytes play a crucial role in maintaining bodily fluid and temperature homeostasis (Miyata, 2022).

Eide et al. (2021) used magnetic resonance imaging to demonstrate that SD may affect human molecular clearance from the brain. The glymphatic system mainly clears toxic substances through astrocytic AQP4-dependent (Verkman et al., 2006; Mestre et al., 2018) circulation of CSF (Iliff et al., 2012) to help neurons function properly. Astrocytic AQP4 regulation of CSF distribution and clearance rate follows the sleep rhythm. It has been widely reported that SD impairs the glymphatic system due to the accumulation of neurotoxic substances (Shokri-Kojori et al., 2018). One proven manifestation is the impairment of AQP4 expression or loss of AQP4 localization (Zeppenfeld et al., 2017). Furthermore, some SD patients have been reported to carry genetic mutations in the AQP4 gene (Iliff et al., 2012). Glymphatic fluid also plays an important role in the delivery and distribution of various substances in the brain (Achariyar et al., 2016). Exploring dynamic subcellular AQP4 relocalization (Salman et al., 2022) and developing astrocytic AQP4 agonists to target clearance or delivery of substances (Achariyar et al., 2016) to different brain regions, allowing recovery of their functions, could be new treatment avenues in the future. Improper astrocytic AQP4 expression results in BBB impairment, leading to weakened brain defenses (Jeon et al., 2021), along with a series of astrocytic AQP4 physiological functions (Nagelhus and Ottersen, 2013) being affected as well. CD44 is a key factor in the regulation of BBB function. SD-induced excessive CD44 expression in hippocampal tissue astrocytes increase BBB permeability, resulting in cognitive impairment (Sun et al., 2020). Recent research has shown that SD causes central inflammation via the activation of astrocytes and microglia (Chennaoui et al., 2015; Bellesi et al., 2017; Xue et al., 2019), as well as an increase in peripheral inflammatory markers, which may occur through the mediation of the gut microbiota-inflammation-brain axis (Wang Z. et al., 2021) and the lung inflammation-brain axis (Mao et al., 2022; Monje and Iwasaki, 2022). Improving the BBB may effectively prevent inflammation from entering the brain, and astrocytes can be used to control the development of inflammation in the CNS. The relationship between astrocytic AQP4 and central inflammatory processes, as well as the mechanisms by which the sleep-wake cycle or SD affects AQP4, should be investigated in future studies.

### 2.4. Neuroinflammation and astrocyte–microglia crosstalk

Accumulating evidence suggests that, as the stromal cells of the brain, astrocytes support tissue-resident immune cells, mainly microglia, which are the resident macrophages (Bohlen et al., 2017; Vainchtein and Molofsky, 2020). Considerable information regarding astrocyte–microglia interactions has emerged in recent years (Liddelow et al., 2020; McAlpine et al., 2021; Rostami et al., 2021; Rueda-Carrasco et al., 2021). In the context of trauma, infection, neurodegenerative diseases, and even SD, diverse responses of microglia and astrocytes can contribute to tissue repair and promote CNS pathology or may exacerbate inflammatory reactions and tissue damage. Atrooz et al. (2019) found that a rise in inflammatory markers is seen in response to chronic SD scenarios, pointing to a possible function for glia in aggravating the injury and prolonging its consequences. The upregulation of glial fibrillary acid protein is the characteristic and common feature of reactive astrocytes in different species, which can be detected at both the protein and mRNA levels. Possibly by modifying the immunological signaling milieu, including the nuclear factor kappa-light-chain-enhancer of activated B cells pathway, calcineurin pathway, mitogen-activated protein kinase pathway, and Janus kinase/signal transducer and activator of transcription 3 pathway, SD can induce pro-inflammatory conditions in the brain (Dumaine and Ashley, 2015; Atrooz et al., 2019; Ensminger et al., 2022). For example, SD may activate the sympathetic nervous system to increase vascular sheer stress causing inflammation. However, a study also revealed that inflammation from SD acts differently, probably independent of the renin-angiotensin system. Bellesi et al. (2017) examined the effects of 6–8 h of sleep, spontaneous awakening, SD, and chronic SD on brain cells. They found that chronic SD potentially induced microglial activation and astrocytic transformation to A1 phenotype, leading to impairment in neuronal synaptogenesis and phagocytosis, and consequently cell death in neurons and oligodendrocytes. The mild, sustained microglial activation and astrocyte reactivity caused by chronic SD resulted in hypersensitivity to secondary attacks, leading to further damage (Bellesi et al., 2017).

Astrocytes are of great importance in the regulation of neuroinflammation (Colombo and Farina, 2016). Disruption of the gene encoding the circadian clock regulator BMAL1 led to significant astrocyte reactivity and inflammation in a C57BL/6 mouse model (Musiek et al., 2013). Astrocytes and tanycytes in the circumventricular organs (CVOs) of the brain are involved in initiating lipopolysaccharide (LPS)-induced inflammatory responses via toll-like receptor 4 (Miyata, 2022).

Astrocytes and microglia can act in concert to phagocytose damaged cellular elements, thus contributing to a healthy homeostasis in the brain, including the suprachiasmatic nucleus (Damisah et al., 2020). Damisah at al. (2020) demonstrated that, in this process, astrocytes will polarize rapidly and engulf many small dendritic apoptotic bodies, and microglia will migrate and engulf the apical dendrites and soma. Astrocytes and microglia are also involved in neuroinflammation-induced neuronal death. IL-1β and TNF-α can trigger the overproduction of astrocyte-derived nitric oxide, leading to neuronal death (Waxman, 2003; Calabrese et al., 2007; Colombo et al., 2012) by facilitating the activation and translocation of NF-κB into the nucleus (Qian et al., 2007). Similarly, microglia contribute to the excessive release of glutamate by astrocytes via the stromal cell-derived factor 1-CXCR4-TNF-α chemokine pathway, thus inducing neuronal excitotoxicity and apoptosis (Bezzi et al., 2001). By secreting IL-1, TNFα, and complement component 1q, microglia-induced neurotoxic A1 astrocytes cause neuronal death and worsen the progression of neurodegenerative illnesses such as AD, HD, and PD (Liddelow et al., 2017; Xu et al., 2018). If synaptic connections do not receive sufficient support from neurotoxic astrocytes, circuit dysfunction may worsen (Liddelow et al., 2017). Additionally, microglia-induced A1 astrocytes can kill oligodendrocytes by secreting toxic lipids (Guttenplan et al., 2021).

Increased expression of the tyrosine-protein kinase MER (MERTK) protein and lipid peroxidation activate astrocytic phagocytosis (Chung et al., 2013, 2015), which plays a critical role in the synaptic remodeling underlying neural circuit refinement. Notably, MERTK is also expressed in microglia (Chung et al., 2013), and structural changes in the MERTK pathway can become apparent after SD or long-time wakefulness (Bellesi et al., 2015). Notably, it has been reported that caffeine and modafinil ameliorated SD-induced neuroinflammation and emotional stress and partially reversed the morphological structure [reactive state of astrocytes can be described with abnormal hypertrophy as well as hyperplasia, and increased expression of GFAP in injury and diseases (Bennett and Viaene, 2021)] of astrocytes and microglia by mediating microglial activation (Wadhwa et al., 2018). Astrocytes and immune cells are known to interact dynamically under neuroinflammatory conditions; however, the extent to which SD-induced neuroinflammation might result in such interactions is unclear. Standardized scales will be required to provide predictions and estimate the degree of neural inflammatory changes in the future.

### 2.5. Sexual difference in astrocytes

Female and male brains differ in their susceptibility to neurologic disorders; patients with multiple sclerosis and Alzheimer’s disease are more likely to be females (Seshadri et al., 1997; Westerlind et al., 2014), while those with intellectual disability, autism spectrum disorder, and Parkinson’s disease are more likely to be males (Werling and Geschwind, 2013; Gillies et al., 2014). Recently, multiple findings identified sex differences in microglia, but sex differences in astrocytes remain elusive despite their extensive interactions with microglia. There is an incomplete list of genes preliminarily representing the evidence of sexual dimorphism in human cortical astrocytes (Krawczyk et al., 2022), but sex differences in astrocyte regulation of sleep are also rarely reported. Sex differences in sleep disorder-related diseases should be further discussed at the genetic level.

## 3. Role of astrocytes in SD-comorbid brain disorders

**FIGURE 3:**
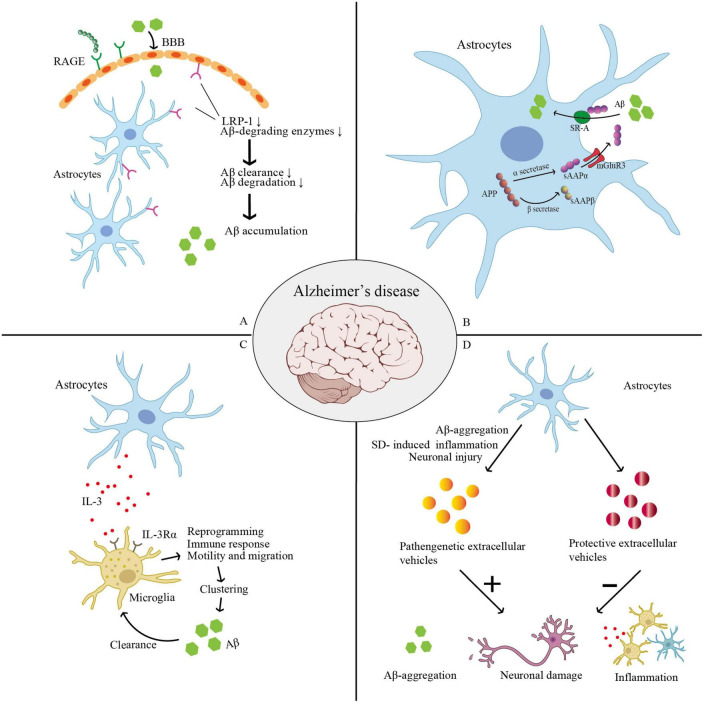
Thess pictures show roles of astrocyte in AD. **(A)** SD leads to low levels of LRP-1 at the BBB and increased expression of RAGE. RAGE may encourage an influx of peripheral Aβ into the brain across the BBB from the peripheral venous circulation. Low levels of LRP-1 reduce Aβ-degrading enzymes, and finally lead to reduced cellular Aβ absorption and degradation. **(B)** APP can be cleaved into sAPPα and sAPPβ in astrocyte. sAPPα can enhance Aβ phagocytosis via activating the SR-A, while sAPPβ can decrease the expression of sAPPα. SD was shown to increase the expression of sAPPβ, in which SD may worsen AD progression. **(C)** Microglia increase their IL-3Rα (IL-3’s particular receptor). Astrocytes naturally release IL-3, which causes microglia to undergo transcriptional, morphological, and functional reprogramming, giving them the ability to cluster and remove Aβ and tau aggregates as well as an acute immune response program. **(D)** Astrocyte-derived extracellular vesicles (ADEVs) can have both pathogenic and protective effects in AD. When subjected to Aβ aggregation, SD-induced inflammatory response, or neuronal damage, astrocytes can release pathogenic extracellular vesicles, which further aggravate Aβ deposition, promote inflammation, and cause loss of neurons and synapses, thus triggering a vicious cycle and promoting AD progression. Astrocytes can release protective EVs to carry a variety of molecular mediators that can inhibit the accumulation of Aβ, promote neuronal survival and synaptic growth, and thus prevent and delay AD progression. (AD, Alzheimer’s disease; APP, amyloid precursor protein; BBB, blood–brain barrier; LPR-1, low-density lipoprotein receptor-related protein 1; RAGE, receptors of advanced glycation end products; sAPPα, soluble amyloid precursor protein; SR-A, class-A scavenger receptor; SD, sleep deprivation).

Lack of sleep or SD is a major comorbidity of neurodegenerative diseases (Kwon and Koh, 2020; Upadhya et al., 2020; Li et al., 2021) as well as pain-related conditions and several other brain disorders such as epilepsy (Li et al., 2019) and mood disorders (Cao et al., 2013; Hines et al., 2013). Moreover, SD may accelerate the development and progression of these diseases, and the breakdown of glial cell homeostasis is the primary cause of neuroinflammation in several neurological disorders. When astrocytes acquire the A2 phenotype, they appear to act protectively; however, when they develop hyperplastic bodies, they are speculated to act as saboteurs (Pekny and Pekna, 2014; Kwon and Koh, 2020). Controlling astrocyte subsets and functions may be a novel strategy to regulate sleep and control the progression of these diseases. Other factors, such as age (Yuan et al., 2021), and distinct brain regions (Jha et al., 2022) alter astrocyte function in certain diseases and cannot be disregarded either (Figure 3).

### 3.1. Alzheimer’s disease

Alzheimer’s disease is a multifactorial disease that causes dementia associated with memory loss, progressive cognitive impairment, and certain abnormal behaviors like aggression and agitation. It is characterized by insoluble neurofibrillary amyloid plaques, hyperphosphorylated tau tangles, aberrant mammalian target of rapamycin activity (Rapaka et al., 2022), and neuronal and synaptic loss, particularly in the hippocampus. Pericytes, astrocytes, vascular endothelial cells, and tight junctions are the BBB structural elements associated with AD pathogenesis.

Several behavioral symptoms and pathological signs of AD have been demonstrated to worsen in response to persistent SD in transgenic mouse models of AD (Zamore and Veasey, 2022). Conversely, sleep enhancement may have a neuroprotective effect. Total amyloid β-protein (Aβ) levels in the hippocampal interstitial fluid were shown to be higher than usual following spontaneous arousal or short-term (6 h) SD, and a similar result was observed in clinical experiments (Kang et al., 2009; Shokri-Kojori et al., 2018). Moreover, a randomized controlled study found that SD altered amyloid levels and tau phosphorylation, suggesting a possible mechanism by which SD increases the risk of AD (Barthélemy et al., 2020).

Here, we outline numerous ways in which astrocytes participate in slowing the course of AD:

(1) Astrocytic transcytosis of Aβ (Domínguez-Prieto et al., 2018). Astrocytes express high levels of low-density lipoprotein receptor-related protein 1 (LRP-1). Accumulating preclinical studies show that LRP-1 not only controls how Aβ is metabolized in the brain and peripheral tissues, but also maintains brain homeostasis, which is likely impaired and contributes to AD development in Aβ-independent ways (Shinohara et al., 2017). SD leads to low levels of LRP-1 at the BBB (Cai et al., 2018) and increased expression of receptors of advanced glycation end products (RAGE) in the hippocampus and prefrontal cortex, and these changes significantly correlate with the transport and removal of Aβ42, a core CSF biomarker for AD diagnosis (Zhao et al., 2019). Liu et al. (2017) show that, in primary astrocytes, LRP-1 knockdown reduced cellular Aβ absorption and degradation. Several significant Aβ-degrading enzymes, including the matrix metalloproteases MMP2 and MMP9, and insulin-degrading enzymes were also downregulated in astrocytes after LRP-1 was silenced. Moreover, conditional *Lrp1* gene deletion in astrocytes from APP/PS1 mice resulted in poor brain Aβ clearance, increased Aβ accumulation, and accelerated amyloid plaque formation without impacting Aβ production. By modulating various Aβ-degrading enzymes and cellular pathways, astrocytic LRP-1 (Liu et al., 2017) and LRP-4 (Zhang et al., 2020) have been shown to be potential therapeutic targets for controlling Aβ clearance in AD (Figure 3A).

(2) The astroglial subtype of metabotropic glutamate receptor 3 (mGlu3R) has neuroprotective effects in the AD course, which are dependent on the secretion of soluble amyloid precursor protein (sAPPα). Astrocytes express sAPPα, which can enhance Aβ phagocytosis by activating the class-A scavenger receptor (SR-A). Durand at al. determined that SR-A mediates mGlu3R- or sAPPα-induced Aβ uptake, and sAPPα is the enhancer of SR-A-dependent Aβ phagocytosis in the process of Aβ clearance by astrocytes. This was proposed as a novel pathway for Aβ clearance (Durand et al., 2019). However, SD was shown to increase the expression of both sAPPβ and β-site APP-cleaving enzyme 1 in the hippocampus and prefrontal cortex while decreasing the expression of sAPPα (Zhao et al., 2019), which undoubtedly worsens AD progression (Figure 3B).

(3) Astrocytic AQP4: SD impairs the glymphatic system by disrupting the normal function of astrocytic AQP4-dependent CSF (Verkman et al., 2006; Iliff et al., 2012; Mestre et al., 2018), leading to BBB impairment, as well as the accumulation and aggregation of neurotoxic substances like α-synuclein and Aβ.

(4) In the context of SD, activated microglia and microglia-activated astrocytes mediate neuroinflammation (Park et al., 2021): various types of damage caused by inflammation can lead to increased degradation of α-synuclein and Aβ. Some possible mechanisms include increased glutamate generation induced by pro-inflammatory substances (such as TNF-α) (Santello and Volterra, 2012), leading to brain excitotoxicity and increased intracellular Ca^2+^ concentration promoting glutamate, ATP, and GABA release (Parpura et al., 1994; Stout et al., 2002; Lee et al., 2010). These processes are also involved in AD pathogenesis (Jo et al., 2014). Thus, establishing reliable therapeutic interventions targeting microglial- and astrocyte-driven molecular pathways in AD progression could be an effective strategy for controlling neuroinflammation (Singh et al., 2020).

(5) Recently, it has been reported that astrocytes and microglia interact to speed up the breakdown of α-synuclein and Aβ, with IL-3 identified as a critical facilitator of this interaction. Microglia increase their IL-3Rα, which is IL-3’s particular receptor, upon recognizing Aβ deposits, making them more receptive to IL-3. Astrocytes naturally release IL-3, which causes microglia to undergo transcriptional, morphological, and functional reprogramming, allowing them to cluster and remove Aβ and tau aggregates and an acute immune response program. Therefore, IL-3-related mechanisms could be a possible target for AD treatment (McAlpine et al., 2021; Rostami et al., 2021). This also reveals the association between astrocyte–microglia cross-talk and the cerebral pathology of AD/PD (Figure 3C).

(6) Alzheimer’s disease patients show dysregulation in sleep, which can be regulated by the orexin system. Orexins promote wakefulness and shorten the duration of REM and NREM sleep; they can also inhibit Aβ clearance, phagocytosis, and autophagic flux in microglia (An et al., 2017; Jones, 2020). Astrocytes regulate the release of orexins (Burt et al., 2011), which have been proposed to play a role in both AD and SD. Moreover, orexins upregulated the Aβ level in the brain interstitial fluid after SD, which was reversed after the infusion of a dual orexin receptor antagonist (almorexant) (Kang et al., 2009). Although orexins and AD are known to be closely associated, orexin levels vary greatly between AD patients. Thus, detecting the releasement of orexin and sleep state may be a supplementary means to keep track of the AD patient’s progress (Liguori et al., 2020; Um and Lim, 2020; Treu and Plante, 2021).

(7) Sleep deprivation can increase astrocytic extracellular ATP concentrations (Schmitt et al., 2012), and ATP is hydrolyzed to adenosine, which regulates sleep as well as synaptic plasticity, cognitive function, information processing, and memory consolidation (Gomez-Castro et al., 2021). Therefore, adenosine receptor A2AR has been suggested as a promising candidate molecule to interfere with the astrocytic ability to regulate synaptic function and memory in rodent models and patients with AD (Matos et al., 2015; Liu et al., 2019). A recent study also indicated that A2AR can regulate dynamic Ca^2+^-related changes in astrocytes via the intertwined P2 × 7R-/P2Y1R-mediated mechanism, which has been shown to be disrupted in early AD, leading to abnormal information processing (Dias et al., 2022).

(8) Biphasic modulation of astrocyte-derived extracellular vesicles (ADEVs) in AD progression (Li B. et al., 2023). Specific conditions and stimuli regulate ADEV number and characteristics so that they can have both pathogenic and protective effects in AD. When subjected to severe stimulation, such as Aβ aggregation, chronic inflammatory response, or neuronal damage, astrocytes can release pathogenic extracellular vesicles, which further aggravate Aβ deposition, promote inflammation, and cause loss of neurons and synapses, thus triggering a vicious cycle and promoting AD progression. When subjected to unstimulated or mildly controlled stimuli, astrocytes can release protective EVs to carry a variety of molecular mediators that can inhibit the accumulation of Aβ, promote neuronal survival and synaptic growth, and thus prevent and delay AD progression. Li B. et al. (2023) summarized two basic ideas for the clinical application of ADEVs: (i) preventing pathogenic ADEVs from being secreted and (ii) encouraging astrocytes to produce and secrete protective extracellular vesicles *in vivo* or *in vitro* and even direct loading of therapeutic cargo onto extracellular vesicles (Figure 3D).

(9) Dysregulation of glycolytic metabolism in astrocytes caused synaptic damage in AD mice, which in turn caused cognitive and behavioral abnormalities. This synaptic damage may have been brought on by reduced I-serine synthesis in the glycolytic branch. Studies have also demonstrated that I-serine supplementation can be used to treat AD (Le Douce et al., 2020).

### 3.2. Parkinson’s disease

Parkinson’s disease, the second most common neurodegenerative disease worldwide, is a progressive neurodegenerative disease influenced by environmental variables and genetic predisposition. PD is characterized by the accumulation of α-synuclein, formation of Lewy bodies and neurites, progressive degeneration of dopaminergic neurons in the substantia nigra pars compacta, and presence of motor and non-motor symptoms, including hyposmia, autonomic dysfunction, depression, and sleep disturbances. A growing body of evidence indicates that sleep loss occurs in the early stage of PD and is a risk factor that may accelerate the course of the disease and increase the possibility of long-term cognitive decline (Mantovani et al., 2018; Maggi et al., 2021). The emerging consensus is that glial cells also participate in the progression of PD (Miyazaki and Asanuma, 2020) through the glymphatic system. In addition, Rostami et al. (2021) discovered that in PD, astrocytes can stimulate the production of MHCII and costimulatory molecules necessary for T-cell activation. When human astrocytes and α-synuclein are co-cultured, both increased MHCII expression and molecules on costimulatory T-cells required for T-cell activation can be found. These results are intriguing for the field of PD, although no functional studies have been carried out to test if these astrocytes may successfully activate T-cells by creating an immunological synapse (Sutter and Crocker, 2022).

As mentioned previously, the glymphatic system is involved in α-synuclein clearance, thus minimizing the pathological damage in PD (Sundaram et al., 2019; Cui et al., 2021; Scott-Massey et al., 2022). Conversely, the inflammatory response and AQP4 deficiency-induced permanent damage to the glymphatic system in SD may lead to PD deterioration.

Non-neuronal cells should be considered when analyzing PD-linked mutations that cause pathogenesis and disease progression, and astrocytes are crucial participants in this process. Leucine-rich repeat kinase 2 (LRRK2) mutations are the most common cause of familial PD. They increase LRRK2 activity, which impairs alpha-synuclein breakdown in neurons and may indirectly affect PD development (Alessi and Sammler, 2018). In addition, an LRRK2 mutation was reported to affect endo-lysosomal capacity in astrocytes and astrocyte uptake or internalization of α-synuclein (Streubel-Gallasch et al., 2021). Chronic SD exposure has recently been demonstrated to worsen genetically predisposed dopaminergic dysfunction in LRRK2 G2019S mice, which is associated with a-synuclein aggregation in the brain and irregular sleep patterns (Liu X. et al., 2022). Therefore, LRRK2-targeted therapies benefit this type of PD, and small-molecule LRRK2 kinase inhibitors are considered highly neuroprotective (Tolosa et al., 2020). Autosomal recessive mutations in the glucocerebrosidase gene, Beta-glucocerebrosidase 1 (GBA1), can induce Gaucher’s disease, a lysosomal storage disorder. The GBA gene encodes the lysosomal enzyme glucocerebrosidase, which maintains glycosphingolipid homeostasis. Heterozygous carriers of most GBA1 mutations have shown a significant increase in PD incidence. Mutations in the GBA gene can lead to loss of glucocerebrosidase activity and lysosomal dysfunction, potentially impairing alpha-synuclein metabolism. Given the crucial role of lysosomal dysfunction in PD pathogenesis, the interaction between GBA1 and LRRK2 has gained attention, as both are enriched in astrocytes (Cahoy et al., 2008). Evidence shows that LRRK2 inhibition may repair lysosomes and inflammatory abnormalities caused by astrocytic GAB1 mutations (Sanyal et al., 2020). Moreover, a study showed that GAB1 mutations strongly increase the risk of REM sleep behavior disorder (Gelegen et al., 2022). However, there are few reports on susceptibility to PD in carriers of GAB1 gene mutations in SD. Nevertheless, it is clear that, as important participants in these processes, astrocytes have great value as therapeutic targets (Wang C. et al., 2021), even in the context of SD.

### 3.3. Pain and mood disorders

#### 3.3.1. Pain

Sleep deprivation is associated with increased pain sensitivity in mice and is a risk factor for clinical pain (Alexandre et al., 2017). Persistent pain seriously affects a patient’s quality of life, as it is associated with a series of comorbidities, including depression, cognitive decline, anxiety, and SD. SD also enhances the response to pain in the primary sensory areas of the cerebral cortex while impinging on the activity in other areas that regulate pain processing, such as the striatum and insula (Krause et al., 2019). Preoperative SD can also amplify the difficulties in managing postoperative pain. Research has demonstrated that neuronal activity and functional connectivity are suppressed in the nucleus accumbens (a subregion of the ventral striatum) and ventrolateral periaqueductal gray (Guo et al., 2022). Furthermore, a recent study found that SD changed pain thresholds and oxidative stress indicators in healthy males, whereas recovery sleep could raise pain thresholds and reverse the effects of oxidative stress on the body (Chen et al., 2022). Notably, in another study, SD was reported to increase pain sensitivity and pain complaints accompanied by an overnight mood improvement (Kundermann et al., 2008). In healthy people with mild sleepiness, extended bedtime may lead to increased sleep duration and decreased drowsiness, which lowers their susceptibility to pain (Roehrs et al., 2012). The medial prefrontal cortex (mPFC) is closely associated with sleep: in mice, SD alters up to 12 circadian core clock genes in the mPFC (Guo et al., 2019); in response to acute SD, microglia in mouse prefrontal brains become active (Liu H. et al., 2022), triggering inflammatory responses that can also result in oxidative stress and lower glutathione levels. Moreover, clinical data indicated that SD alters the anatomy of the mPFC in humans (Feng et al., 2018). Finally, an animal study of adult male Sprague-Dawley rats showed that the mPFC also plays a role in pain (Chen et al., 2022). As stated above, alternative astrocytic pathways have not yet been investigated except for neuroinflammation-induced hyperalgesia.

**FIGURE 4:**
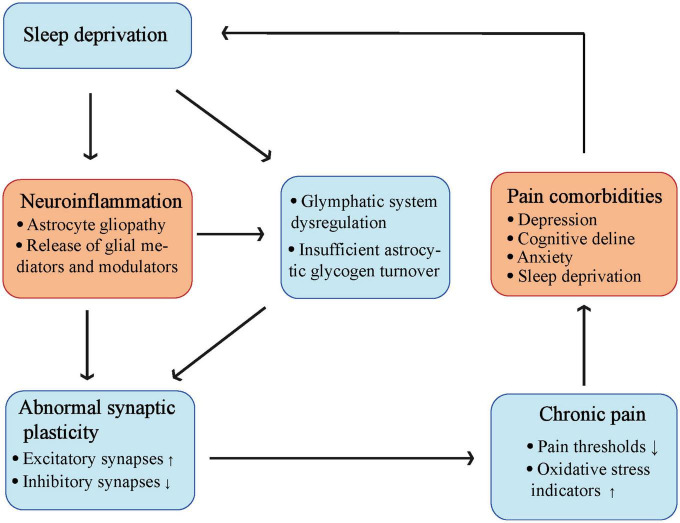
The complicate interaction between sleep deprivation and chronic pain. Sleep deprivation can cause activation of astrocytes, and release of glial mediators as well as modulators to induce neuroinflammation. Both sleep deprivation and neuroinflammation can lead to glymphatic system dysregulation and insufficient astrocytic glycogen turnover. These changes finally cause abnormal synaptic plasticity leading to chronic pain, make a vicious circle into pain comorbidities including sleep deprivation (Created with BioRender.com).

Migraines may also be associated with glial cell activation (Bartley, 2009). It has been hypothesized that one of the causes of migraine and depression may be SD-mediated insufficient astrocytic glycogen turnover (Bellesi et al., 2018) that makes the brain more susceptible to cortical diffusion depolarization owing to the impaired extracellular clearance of potassium and glutamate (Petit et al., 2021; Del Moro et al., 2022). Importantly, sleep regulates the glymphatic system (Xie et al., 2013), which is a brain fluid transport system that clears proteinaceous waste. The brain may be unable to eliminate these waste products due to glymphatic dysfunction caused by astrogliopathy, which may result in the accumulation of neuroinflammatory mediators and persistent pain. Thus, studies aimed at addressing the role of glymphatic function and dysfunction in pain remain an important direction for future research. It is important that sleep should be considered as a targeted therapy in clinical and external hospital pain regulations. In addition, antioxidant and anti-inflammatory pathways also contributed to the regulation of SD-induced hyperalgesia (Figure 4).

#### 3.3.2. Mood disorders

Mood disorders refer to a range of diseases, including major depressive and bipolar disorders, that can lead to recurrent, chronic, and disabling tendencies (Wittchen, 2012; Palagini et al., 2022). SD and rhythm changes are core symptoms of these diseases in almost all patients, and it has been reported that SD has a complex bidirectional relationship with mood episodes (Geoffroy, 2018). The antidepressant effects of SD have been previously studied (Kundermann et al., 2008; Trautmann et al., 2018), and astrocytes were found to function by activating medial prefrontal cortex P2 × 2 receptors and synaptic adenosine (A1) receptors (Hines et al., 2013), causing adenosine-mediated antidepressant-like effects (Cao et al., 2013). In contrast, SD may be a risk factor for mood disorders in healthy individuals (Ablin et al., 2013). Rats exposed to SD have altered prefrontal cortical 5-reductase expression and activity, impacting their mental health (Frau et al., 2017). After SD, microglia and astrocytes mediate neuroinflammation, which may predispose patients to neuropathic progression in mood disorders by dysregulating feed-forward on the hypothalamic-pituitary-adrenal axis (Palagini et al., 2019), causing abnormal synaptic pruning, early synaptic loss, and neurodegeneration (Madore et al., 2020). Specifically, by generating pro-inflammatory substances, SD may directly contribute to neuroinflammation and mediate glutamate-mediated excitotoxicity and neuronal damage by activating microglia through abnormal pruning and altering astroglia-neuron signaling. These processes may then amplify the neuroinflammatory effects of neurodegeneration, promoting mood disorders, cognitive dysfunction, and memory impairment. Since this involves negative glycogen turnover and impaired glutamatergic neurotransmission through the ANLS, we may find a promising method to interrupt the vicious cycle of sleep loss and improve mood disorders. Another reported pathway of astrocyte-associated SD-induced depression involves the activation of astroglial P2 × 7 receptors by SD, triggering depression-like behaviors and selectively downregulating astrocytic 5-HT2B receptors. P2 × 7R-knock-out mice showed alleviated depression-like behaviors, suggesting that 5-HT2B receptors can play a key role in targeted therapies aimed at SD-induced depression (Xia et al., 2020). In addition, activated leucine-rich repeat protein-3 (NLPR3) inflammasomes in astrocytes can decrease brain-derived neurotrophic factor (BDNF) levels. This is reported to be a crucial pathological event in depression-like behavior after SD. Moreover, leptin has a synergistic effect on the antidepressant effects of fluoxetine by increasing the expression of astroglial 5-hydroxytryptamine receptor 2B (5-HT2B), which enables fluoxetine to amplify the level of astrocyte-derived BDNF (Li et al., 2018).

### 3.4. Other diseases

#### 3.4.1. Traumatic brain injury

In both the cerebral cortex and hippocampus, activation of astrocytes can mediate abundant astrogliosis, leading to increased levels of the astrocytic marker GFAP after mild traumatic brain injury (TBI) as well as a negative effect on the brain (Hazra et al., 2014; Sabir et al., 2015). Astrocyte-derived GFAP in blood has been reported to predict death after severe TBI (Vos et al., 2004). SD may reprogram the transcriptome of genes involved in plasticity, neuroprotection, and circadian rhythms in a way detrimental to brain recovery after mild TBI, especially in the cerebral cortex. In fact, SD has been shown to amplify the damage caused by mild TBI, with an increase in astrocyte-derived GFAP (Sabir et al., 2015), although astrocytes have mutually contradictory effects on TBI. For example, they accelerate and suppress neuroinflammation, promote and restrict neurogenesis and synaptogenesis, and disrupt and repair the BBB through multiple regulatory molecules. Although these effects have not been shown to be associated with SD, astrocytes are novel and attractive targets for therapeutic drugs for TBI (Michinaga and Koyama, 2021).

#### 3.4.2. Stroke

Sleep deprivation can be a preexisting risk factor and direct consequence of brain damage, such as stroke (Pérez-Carbonell and Bashir, 2020). Astrocytes play a mixed role in stroke pathology—the activated subtype provides neuroprotection while secreting inflammatory molecules that aggravate stroke damage. Astrocytic AQP4 also has a complex bimodal function in stroke pathology: knockdown or inhibition of AQP4 in astrocytes can mediate protective effects (Hirt et al., 2017; Wang et al., 2020) or cause damage (Shi et al., 2012; Zeng et al., 2012). Although their mechanisms of action remain incompletely understood, the damage and repair effects of astrocytes in stroke pathology make them a significant target for stroke treatment (Patabendige et al., 2021). Whether improving sleep helps limit stroke damage remains to be investigated.

#### 3.4.3. Epilepsy

Nighttime epilepsy can seriously affect sleep quality, and in turn, SD or the side effects of antiepileptic drugs can promote the progression of epilepsy. The waste disposal assumption explains how epilepsy causes astrocytes to deal with extracellular glutamate and K^+^, leading to random neuronal hyperexcitability (Rabinovitch et al., 2019). Similarly, it is hypothesized that SD-induced negative glycogen turnover in astrocytes leads to a disorder in extracellular K^+^ recycling, which may be a possible causative risk for epilepsy (DiNuzzo et al., 2015). Furthermore, ADK in astrocytes alters the inflammatory microenvironment in the epilepsy brain (Aronica et al., 2013). Nevertheless, it is still unclear how SD and ADK expression in astrocytes are related.

### 3.5. Circadian dysfunction and astrocytes

Endogenous circadian rhythms underlie neurobehavioral processes, including physiological alertness and cognitive functioning. The sleep homeostat and circadian drives are the two mechanisms that regulate sleep (Saper et al., 2005). Circadian dysfunction can lead to sleep deprivation, causing a range of behavioral effects. Both acute and chronic SD can increase homeostatic sleep drive and worsen waking neurobehavioral functions, which are reflected in drowsiness, attention, cognitive speed, and memory.

In recent years, a series of reports have shown that genes in astrocytes regulated by the circadian rhythm play a role in neurological diseases. Neuroinflammation is an important component of many neurological diseases. Following REM sleep deprivation, studies have reported an elevated expression of BMAL1 and Per2 proteins, along with reduced Egr1 protein expression in the hippocampus. This alteration is accompanied by the activation of reactive astrocytes, which contribute to neuroinflammatory impairments (Hou et al., 2019). Hou et al. (2019) claimed that circadian genes *Bmal1*, *Per2*, and *Egr1* participate in the SD-related aggravation of hippocampal neuroinflammatory impairments that activate reactive astrocytes; however, the underlying mechanism remains unclear. The core clock protein BMAL1 serves as the primary positive circadian transcriptional regulator. In a C57BL/6 mouse model, disruption of the gene encoding the circadian clock regulator BMAL1 resulted in significant age-related astrogliosis and inflammation (Musiek et al., 2013). Another circadian clock protein, Rev-erbα, a nuclear receptor and circadian clock component, can mediate microglial activation and inflammation. In the hippocampus, Rev-erbα deletion caused spontaneous microglial activation and increased the expression of proinflammatory transcripts by increasing basal NF-κB activation and secondary astrogliosis. According to this study, the circadian clock and neuroinflammation are linked by Rev-erb, which is pharmacologically available (Griffin et al., 2019).

Recent studies have linked BMAL1 to protein degradation in astrocytes within the brain. McKee et al. (2023) demonstrated that deleting Bmal1, specifically in astrocytes, leads to a unique cell-autonomous activation state. This activation phenotype not only disrupts circadian function but also hampers the supportive role of astrocytes to neurons while simultaneously increasing extracellular protein degradation (McKee et al., 2023). *In vitro*, increased endocytosis, lysosome-dependent protein cleavage, and an accumulation of LAMP1- and RAB7-positive organelles (which show increased lysosomal abundance) are all seen in Bmal1-deficient astrocytes. *In vivo*, using electron microscopy, astrocyte-specific *Bmal1*-knockout brains exhibit an accumulation of autophagosome-like structures. While it is unclear how BMAL1 influences these astrocyte functions, McKee et al. (2023) claim that *Bmal1* deletion affects several genes related to the endolysosomal system.

## 4. Potential interventions targeting astrocytes to prevent or treat sleep-associated brain disorders

### 4.1. Improving sleep quality

Improving sleep can help relieve the stress associated with disease progression. Sleep health education, exercise (Jurado-Fasoli et al., 2020), music therapy (Tang et al., 2021), light therapy (Brown et al., 2022), melatonin (Zisapel, 2018), dietary habits (Pot, 2018), sleep-induced substance replenishment, and hypnotic drug therapy are common treatments that promote sleep quality.

### 4.2. Regulating neuroinflammation by pharmacological methods

A complex causal relationship exists between diseases, sleep disorders, and neuroinflammation. Breaking any part of this link may stop the vicious cycle and bring benign disease outcomes. Suppression of inflammation is one of the most widely studied therapeutic approaches. Agonists of the α7 nicotinic acetylcholine receptor can activate downstream PI3K/Akt/GSK-3β, restrain the increase of pro-inflammatory factors, and stabilize the expression of anti-inflammatory factors, transcriptor Nrf-2, and antioxidant enzyme HO-1 after SD (Xue et al., 2019). Farnesol, Caffeine and Modafinil can ameliorate SD-induced neuroinflammation and microglial activation (Wadhwa et al., 2018; Li Y. et al., 2023). Sinomenine, or cocculine, is an alkaloid found in the root of the climbing plant Sinomenium acutum, which is now considered a beneficial treatment target for CNS diseases such as AD, PD, depression, TBI, epilepsy, multiple sclerosis, cerebral ischemia, and intracerebral hemorrhage (Hong et al., 2022). Mechanistic investigations have revealed that the anti-inflammatory effects of sinomenine in astrocytes involve the activation of astrocytic dopamine D2 receptors. This activation leads to the nuclear translocation of αB-crystallin (CRYAB), phosphorylation of STAT3, and subsequent inhibition of astrocyte activation (Qiu et al., 2016). It has been reported that Sinomenium can inhibit the production of NO, IL-6, IL-18, IL-1β, TNF-α, and interferon-γ (IFN-γ) in primary cultured human astrocytes and mouse C8D1A astrocytic cell lines and monocyte chemoattractant protein-1 (MCP-1), which was induced by oligomeric Aβ-mediated reactive astrocytes in the brain of patients with AD (Singh et al., 2020). Sinomenium suppresses astrocyte hyperplasia by inhibiting the NLRP3 inflammasome in mouse spinal cord tissue, contributing to the regulation of multiple sclerosis (Kiasalari et al., 2021).

Perioperative neurocognitive disorder (PND) involves brain network disturbances in several different functional brain regions, including brain regions associated with the sleep-wake rhythm, subregions associated with context and fear memory, the septa-hippocampus circuit, the hippocampal-amygdala circuit, and the entorhinal hippocampal circuit. A recent isoflurane-mediated PND animal study showed that enhanced gap junction connexin-43 in astrocytic gap linking could improve long-term isoflurane-induced brain network dysfunction and cognitive impairment by regulating oxidative stress and neuroinflammation. Namely, the gap junction connexin-43-mediated astrocytic network could be the neural circuit underlying the pathological mechanism of PND (Dong et al., 2022).

### 4.3. Potential therapies for targeting astrocytes

A decade ago, the use of induced pluripotent stem cells derived from human somatic cells to treat neurological diseases was widely proposed (Yuan and Shaner, 2013). Astrocyte-targeted therapies for AD aim to prevent the transformation of reactive astrocytes into type A1 astrocytes and encompass strategies such as stem cell therapy, pharmacological interventions, and gene-editing technologies (Bi et al., 2022). In the restorative treatment of PD, co-grafting neural progenitors with engineered astrocytes, which can provide a favorable brain environment for better maturation as well as a better survival rate of the graft by overexpressing the transcription factors *Nurr1* and *Foxa2*, contributes to therapeutic improvement (Tsai, 2018). Currently, there are promising clinical methods for the remote and selective manipulation of astrocytes to correct dysfunction without genetic modification, such as magnetomechanical stimulation. Specially designed magnetic devices are used to determine the mechanosensory threshold of astrocytes and identify submicrometer particles for effective MMS. Specifically, the mechanosensitivity of astrocytes enables placing antibody-functionalized magnetic particles in a magnetic field to trigger mechanogated Ca^2+^ and ATP signaling in astrocytes (Yu et al., 2022).

Inwardly rectifying Kir4.1 channels in astrocytes play a novel role in regulating the expression of BDNF, and inhibition of Kir4.1 channels was found to elevate the expression of BDNF and extracellular glutamate and K^+^ levels at synaptic clefts. These changes may contribute to the elevation of synaptic plasticity and neuronal connectivity and highlight the therapeutic potential of Kir4.1 channels in brain diseases such as depression and epilepsy (Ohno et al., 2021). Furthermore, mesencephalic astrocyte-derived neurotrophic factors have been reported to have great potential for restoring dopaminergic neural circuits. Although limited efficacy has been reported, improved techniques may help us further understand their therapeutic value as neurorestorative agents (Domanskyi et al., 2015). From the perspective of the relationship between astrocyte glycolysis and the course of AD, glucagon-like peptide-1 has been shown to provide neuroprotection in patients, with the underlying mechanism involving activation of the PI3K/Akt pathway, which also provides us with a means to intervene in the progression of AD through astrocytes (Zheng et al., 2021). As stated previously, the inhibitory effect of fluoxetine on the NLRP3 inflammasome in astrocytes is mediated by 5-HT2B receptors, which increase the release of BDNF and have been proven to reduce depression-like behaviors in mice. Leptin enhances the effect of fluoxetine on 5-HT2B receptors by increasing 5-HT expression. All these studies demonstrate the therapeutic value of astrocytes in antidepressants induced by SD (Li et al., 2018).

## 5. Conclusion

Astrocytes have a variety of functions in SD and SD comorbidities that are not yet clearly understood. We aimed to explore better therapeutic interventions for SD comorbidities by elaborating on the specific pathological changes in astrocytes under normal physiological sleep and SD, including those related to ion homeostasis, brain metabolism, the glymphatic system, astrocyte–microglia cross-talk, and neuroinflammation. Additionally, we discussed the potential functions of astrocytes in several SD-comorbid brain disorders, including AD, PD, pain, mood disorders, TBI, stroke, and epilepsy. The findings highlight the need to clarify the complicated roles of astrocytes in the brain under both normal and pathophysiological conditions. Further, we discussed the potential roles of astrocytes in preventing or treating sleep-disorder-related brain diseases. Addressing these issues will open the door to understanding the cellular and circuit processes underlying the pathologies of SD-associated comorbidities.

## 6. Discussion

The high prevalence of SD in daily life reminds us of the necessity to more clearly search for the role of astrocytes in SD and SD-related comorbidity. SD upregulates most of the astrocyte genes, which are associated with metabolism and neuroglial interactions. Furthermore, except for the known genes related to the sleep-wake cycle, further research would investigate the mechanisms underlying how astrocytes act in neural circuits and regulate the brain.

## Author contributions

MQ drafted the manuscript and prepared the figures. GZ, YL, and XW prepared the draft. XL and ZZ revised the manuscript. All authors contributed to the manuscript and approved the submitted version.
